# The Effect of High Protein-High Fat and High Protein-High Carbohydrate Meals on Resting Metabolic Rate and Metabolic Factors in Overweight and Obese Adults: The Study Protocol for a Randomized Crossover Clinical Trial

**DOI:** 10.5812/ijem-157244

**Published:** 2024-10-30

**Authors:** Saber Sahebi, Fatemeh Sadat Hashemi Javaheri, Zahra Valeh, Lida Jarahi, Mohammad Safarian, Mohsen Nematy

**Affiliations:** 1 Department of Nutrition, School of Medicine, Mashhad University of Medical Sciences, Mashhad, Iran; 2 Student Research Committee, Mashhad University of Medical Sciences, Mashhad, Iran; 3 Department of Physical Education, Mashhad Branch, Islamic Azad University, Mashhad, Iran; 4 Department of Community Medicine, Faculty of Medicine, Mashhad University of Medical Sciences, Mashhad, Iran

**Keywords:** Obesity, Protein, Fat, Carbohydrate, Metabolic Factors, Over Weight

## Abstract

**Background:**

The macronutrient composition of daily meals plays a crucial role in influencing the body's metabolic responses during the postprandial phase. However, existing research on the effects of macronutrients, particularly fats and carbohydrates, has produced inconsistent findings.

**Objectives:**

This study aims to evaluate the postprandial effects of two high-protein meals—one low in fat and high in carbohydrates (HP-LF-HC) and the other high in fat and low in carbohydrates (HP-HF-LC)—on energy metabolism, appetite response, and blood markers in overweight and obese men and women without underlying health conditions.

**Methods:**

This study was conducted as an acute randomized crossover clinical trial at the Health Monitoring Center of Mashhad University of Medical Sciences (MUMS) within Imam Reza Hospital, Mashhad, Iran. A total of 30 overweight and obese men and women, meeting the eligibility criteria and free of underlying diseases, were recruited through a public call. Participants were randomly assigned to receive both intervention meals, with a washout period of at least one week between each trial.

**Results:**

The primary outcomes focused on the acute effects of the two dietary interventions on energy metabolism, particularly resting metabolic rate (RMR), and appetite response. Secondary outcomes included changes in lipid profiles, insulin, blood glucose levels, thyroid hormones, and epinephrine.

**Conclusions:**

This study aims to identify which macronutrient composition most effectively enhances resting energy expenditure. The findings could provide valuable insights for dietitians in developing more efficient dietary plans, helping overweight and obese individuals maintain an ideal weight or achieve weight loss by modifying food composition without altering meal volume.

## 1. Background

Historically, obesity and overweight were believed to primarily affect specific demographic groups. However, it is now evident that weight-related issues are widespread (1). By 2025, approximately 167 million individuals are projected to suffer from diseases related to obesity and overweight, as indicated by non-communicable diseases (NCDs) (2). The economic impact of obesity and overweight on gross domestic product (GDP) is escalating, with projections indicating it could reach 3.29% by 2060, particularly in low- and middle-income countries (3).

Recent research suggests that the causes of obesity and overweight extend beyond the simple consumption of excess calories. Factors such as food sources, preparation methods, consumption patterns, and food quality play critical roles in the physiological mechanisms influencing obesity. These factors impact metabolism and weight changes by affecting hormones, altering gut microbiota, and influencing appetite and satiety control centers (4, 5). A common approach to weight control involves consuming low-calorie diets with varying macronutrient compositions (6). While reducing energy intake can lead to weight loss, the body's adaptive mechanisms—such as reduced energy expenditure and preservation of vital energy reserves—can eventually slow or reverse this weight loss. This phenomenon is attributed to a decrease in metabolism and resting metabolic rate (RMR) over time (6, 7).

Most studies report that high-protein meals enhance thermogenesis during the acute phase following meal intake. The energy required for metabolizing protein is approximately four times greater than that for carbohydrates and 7.5 times greater than that for fat (8). However, when examining the effects of fat and carbohydrates, studies yield varying results. Some researchers assert that there is no significant difference between proteins and carbohydrates in diet-induced thermogenesis and thermogenic effects. They suggest that the consumption of high-fat diets may be associated with increased metabolism in obese subjects (9). Conversely, other studies indicate that high-carbohydrate meals exhibit a more pronounced thermogenic effect compared to high-fat meals, with no significant difference observed between normal-weight and obese individuals (10).

In a study conducted by Blundell et al., subjects who typically consumed a high-fat diet exhibited lower energy expenditure compared to those on a low-fat diet. Furthermore, the consumption of a high-fat meal was shown to increase fat oxidation and, consequently, metabolism, while carbohydrate oxidation remained similar in both groups following a high-carbohydrate meal (11).

The existing literature presents conflicting results regarding the impact of macronutrient composition on metabolic adaptation. Therefore, this study aims to evaluate the most effective macronutrient composition for promoting metabolic adaptation and facilitating weight loss.

To ensure homogeneity within our study population, we will include only overweight and obese men and women. Measurements will not be conducted during menstruation. If a female subject is menstruating at a scheduled appointment, the measurements will be postponed to a later, more appropriate time.

## 2. Objectives

The primary objective of this study is to evaluate the effects of two distinct meal compositions—high-protein, high-fat, low-carbohydrate (HP-HF-LC) and high-protein, low-fat, high-carbohydrate (HP-LF-HC) meals—on appetite response (AR), RMR, diet-induced thermogenesis (DIT), and respiratory quotient (RQ) in adults aged 18 to 65.

The secondary objective is to assess the postprandial effects of HP-HF and HP-LF meals on blood glucose, low-density lipoprotein cholesterol (LDL-C), high-density lipoprotein cholesterol (HDL-C), triglycerides (TG), serum insulin, thyroid-stimulating hormone (TSH), thyroxine (T4), and epinephrine.

## 3. Methods

### 3.1. Trial Design

**Figure 1.**
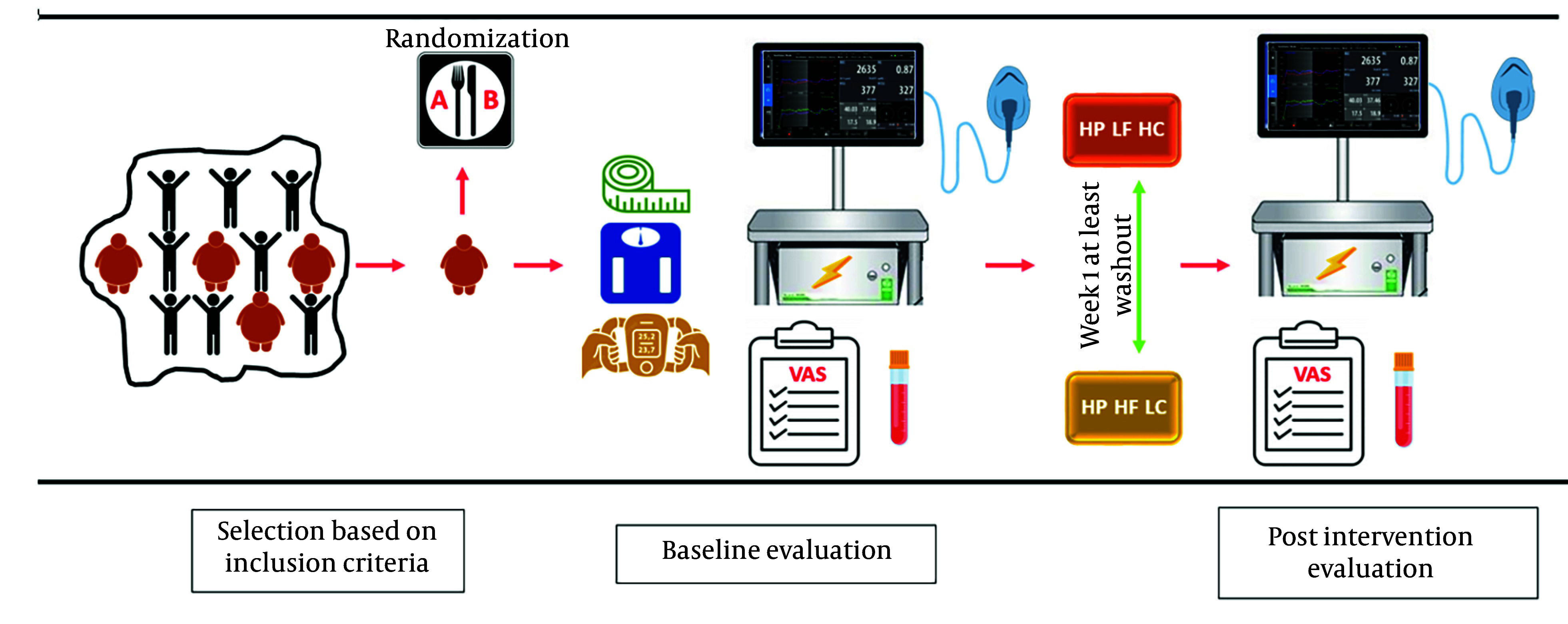
Stages of intervention and evaluation

This study was structured as an acute, randomized crossover clinical trial to evaluate two meals with identical protein content but differing in fat and carbohydrate proportions (HP-HF-LC and HP-LF-HC). Each participant consumed both meals on separate days, with a minimum one-week washout period between sessions to eliminate carryover effects. The stages of intervention and evaluation were outlined in Figure 1. 

Two days prior to the intervention, participants were contacted and instructed to abstain from alcohol consumption, strenuous physical activity, and any dietary changes until their scheduled visit. On the day of the intervention, participants were required to arrive at the Imam Reza Hospital Research Center at 7:00 a.m., using personal vehicles or public transportation while minimizing physical exertion. Participants observed a 12-hour fasting period prior to their arrival, during which they could consume only water and had to refrain from smoking. 

Upon arrival, eligibility for participation was reconfirmed, followed by the measurement of anthropometric parameters. Participants completed an appetite questionnaire and underwent an indirect calorimetry (IC) assessment in a fasting state. Immediately after the IC assessment, a venous blood sample was collected. Participants then consumed the assigned intervention meal within 10 minutes under researcher supervision. 

**Figure 2.**
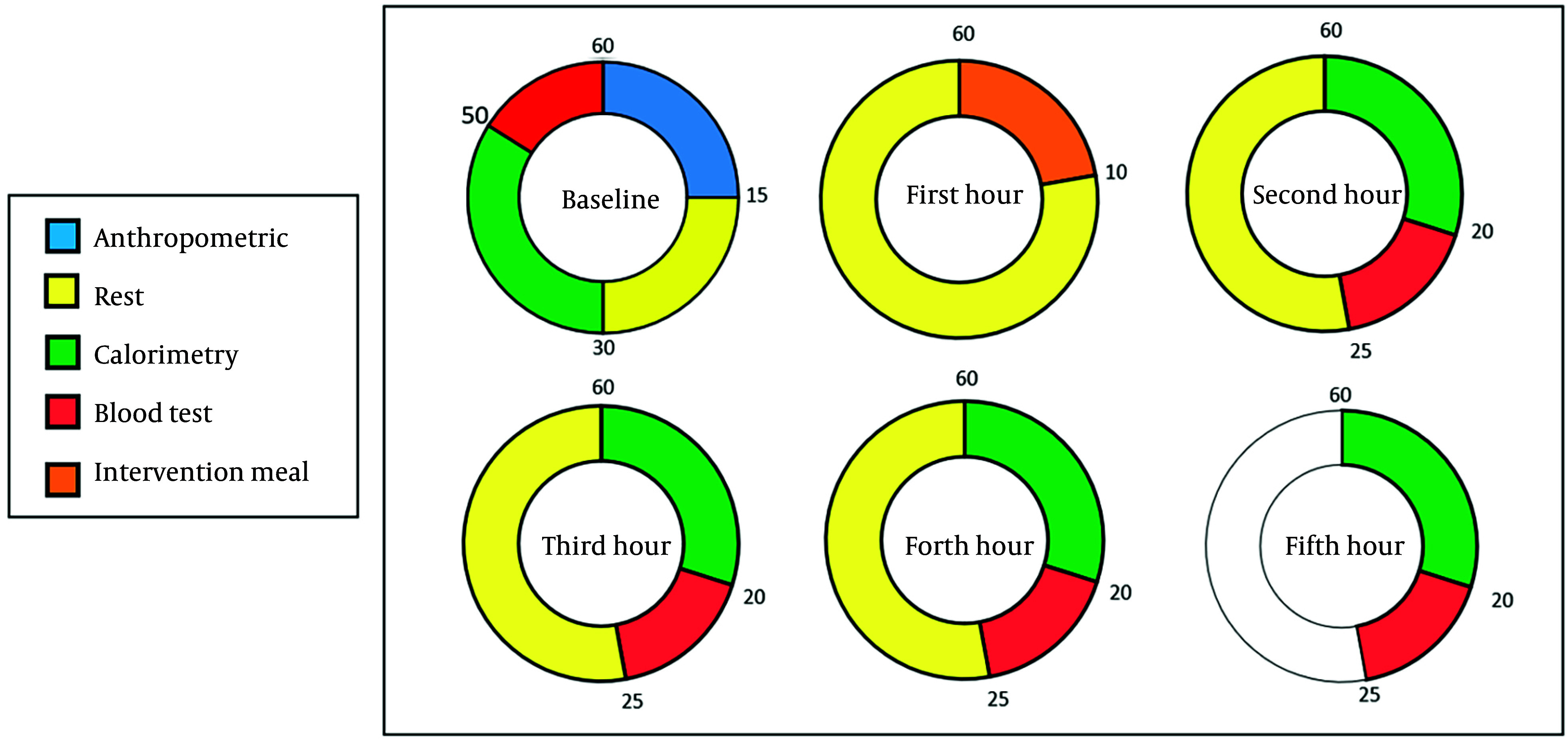
Schedule of the intervention and assessments

During the postprandial phase, IC and appetite response assessments were conducted four times at one-hour intervals. Blood samples were collected twice: One hour after the meal and again during the final hour of the assessment. All tests were performed under standardized conditions and administered by the same personnel to ensure consistency. Figure 2 provided a schedule of the intervention and assessments, with time intervals selected based on prior studies. 

### 3.2. Participants, Interventions and Outcomes

#### 3.2.1. Study Setting

This randomized crossover study was conducted at the Persian Study Research Center, located at Imam Reza Hospital, affiliated with Mashhad University of Medical Sciences (Mashhad, Iran). Participants were recruited through advertisements disseminated via posters placed in healthcare centers across the city and email outreach at Mashhad University of Medical Sciences. The study was retrospectively registered with the Iranian Registry of Clinical Trials (IRCT) under registry number IRCT20240106060633N1.

#### 3.2.2. Eligibility Criteria

##### 3.2.2.1. Inclusion Criteria

The study enrolled 30 adult participants aged 18 - 65 years diagnosed with overweight or obesity, defined by a BMI range of 25 - 40. Eligible participants had not experienced weight changes exceeding 5% in the past three months. Additionally, they had not taken any medications or followed dietary interventions aimed at weight loss within the last 3 - 6 months. Participants were required to refrain from engaging in intense physical activity and were not professional athletes (MET-min < 600 min/week).

##### 3.2.2.2. Exclusion Criteria

Exclusion criteria included: (1) Meal intolerance, (2) the occurrence of adverse health events during the study, (3) engaging in heavy physical activity during the study, (4) tobacco use during the study, (5) unwillingness to continue participation. 

Patient information was recorded, and eligible participants were contacted to receive a detailed explanation of the study plan. On the day of the visit, participants signed a standard written consent form. Those who declined to provide informed consent for any reason were not included in the study. 

#### 3.2.3. Additional Consent Provisions for Data and Specimen Use

Additional consent was obtained for the collection and utilization of data and biological specimens.

### 3.3. Sample Size

Due to the novelty of our study and the absence of prior similar research, the sample size was determined based on the study by Soares et al. (12). The respiratory exchange ratio was selected as the main variable, with the alpha error set at 0.05 and the beta error at 0.2. The sample size was calculated using the two-mean test formula for a quantitative trait in two independent groups. To account for potential attrition, the final sample size was determined to be 30 participants.


$$n=\frac{{\left(Z_{1-\frac{\alpha }{2}}+Z_{1-\beta }\right)}^{2}\left(\sigma_{1}^{2}+\sigma_{2}^{2}\right)}{{\left(\mu_{1}-\mu_{2}\right)}^{2}}$$

### 3.4. Study Protocol and Ethical Approval

The study protocol was approved by the local Institutional Review Board (IRB). Informed consent was obtained from all participants or their legal guardians prior to randomization. The consent form included details about the study, outlined the use of data in the event of participant withdrawal, and granted permission for sharing relevant data with academic experts or regulatory authorities (ethics code: IR.MUMS.MEDICAL.REC.1402.411).

### 3.5. Data Collection at Baseline

At baseline, the demographic and clinical data of the participants were collected.

### 3.6. Interventions

#### 3.6.1. Intervention A

In this intervention, participants received a meal providing 25% of their daily energy requirement. The macronutrient composition of this meal included 25% protein, 45% fat, and 30% carbohydrates.

#### 3.6.2. Intervention B

In this intervention, participants also received a meal providing 25% of their daily energy requirement. However, the macronutrient composition differed, consisting of 25% protein, 15% fat, and 60% carbohydrates. 

To determine daily energy requirements, each participant's RMR was measured using an indirect calorimetry device. Total energy expenditure (TEE) was then estimated by multiplying the RMR by the thermic effect of food and a physical activity coefficient. The meals, prepared in the nutrition department's kitchen, were based on each participant's TEE and composed of ingredients supplied by a contractor. The specific composition of the meals is provided in Table 1.

**Table 1.** Macronutrient and Fiber Composition of Experimental Meals ^a^

Variables	HP-HF-LC Meal 100 (g) ^b^	HP-LF-HC Meal 100 (g) ^b^
**Moisture (%)**	68.88	70.27
**Dry matter (g)**	31.12	29.73
**Protein ** ^**c**^	10.29 (24.97)	8.26 (25.57)
**Carbohydrate ** ^**c**^	12.78 (31.01)	19.42 (60.12)
**Fat ** ^**c**^	8.05 (43.96)	2.05 (14.28)
**Energy (kcal)**	164.8	129.2
**Fiber (g)**	1.64	2.58

Abbreviations: HP-HF-LC, high-protein, high-fat, low-carbohydrate; HP-LF-HC, high-protein, low-fat, high-carbohydrate.

^a^ Values are expressed as gram (percentage of energy).

^b^ The macronutrient and fiber composition in 100 grams of the sample meals has been measured.

^c^ The amounts of macronutrients and fiber are reported in grams on a dry matter basis.

Both meals were identical in terms of ingredients. Participant feedback on the palatability and suitability of the meals was collected using a questionnaire. The quantitative specifications and micronutrient content were pre-determined by the food product manufacturer.

#### 3.6.3. Discontinuing or Modification of Interventions

Given the participants' initial dietary history and routine food intake, no complications were anticipated from the interventions. However, if any complications arose at any stage of the study, the study was promptly halted.

#### 3.6.4. Strategies to Improve Adherence to Interventions

During the registration process, participants were asked about their food preferences, interests, and allergies. The meals provided, which included toast and dairy products, were designed to align with the typical taste preferences of the target population. Individual instructions on meal consumption were given to each participant, and a designated team member was responsible for monitoring proper meal intake.

#### 3.6.5. Interventional Care

While this study was expected to involve minimal complications, participants were restricted from consuming any food or drink other than water during the study and after meal consumption. Potential complications, such as hypoglycemia or issues related to blood sampling, were anticipated, and a physician was present on-site throughout both the interventional and observational stages.

## 4. Results

**Figure 3.**
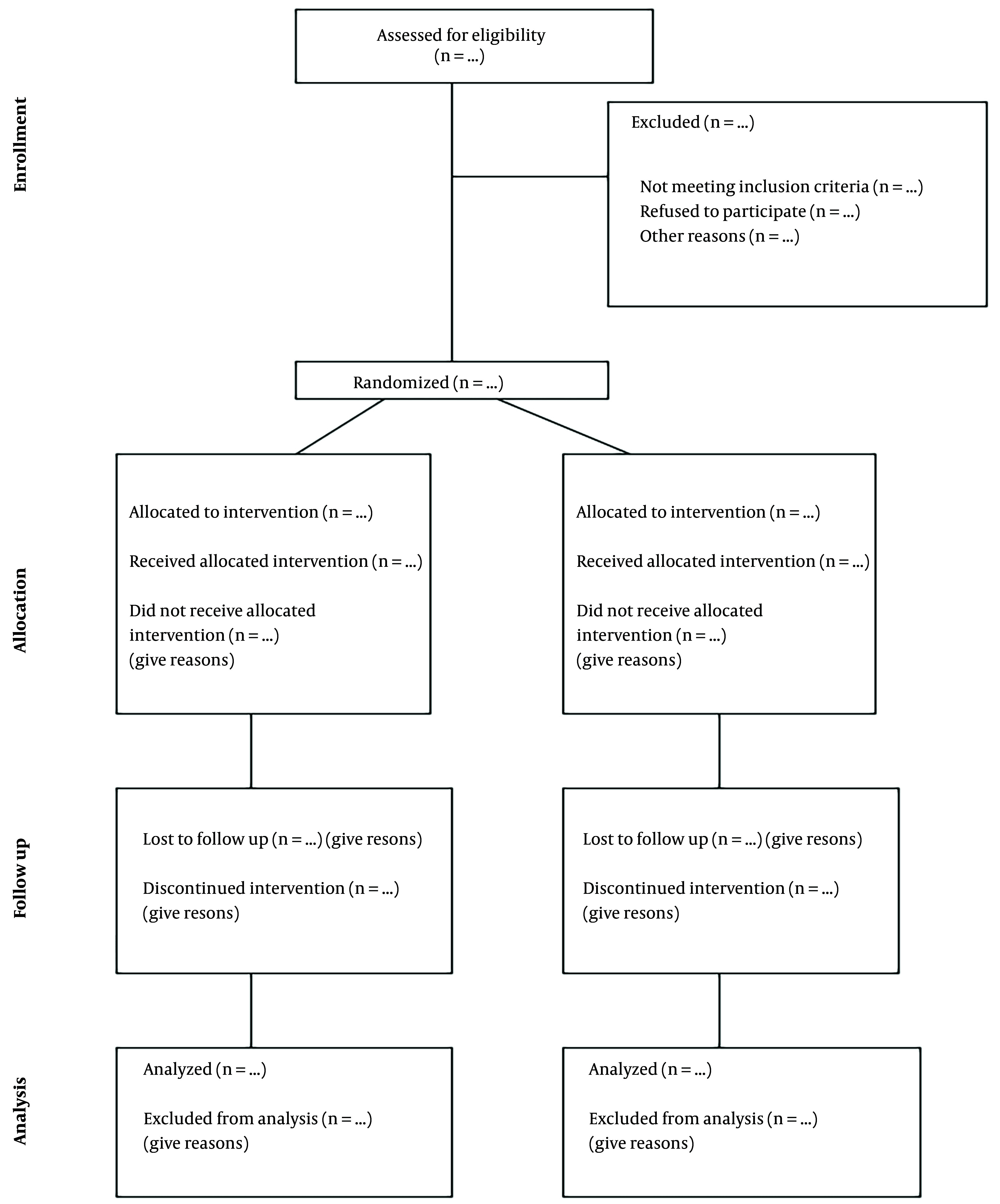
CONSORT diagram illustrates the flow of participants through each stage of a randomized trial

The CONSORT diagram was presented in Figure 3. As this article was a protocol study, it was anticipated that the diagram would be completed upon the study's conclusion. The primary outcome of this crossover trial was to investigate and compare the postprandial acute effects of two different meals on basal metabolic rate and satiety in overweight and obese men and women. The secondary outcome evaluated the impact of these meals on various blood markers, including blood glucose, LDL, HDL, total cholesterol (TC), TG, serum insulin, TSH, T4, and epinephrine.

### 4.1. Assignment of Interventions: Allocation Sequence Generation

This randomization was conducted using a simple randomization technique to allocate participants in a 1:1 ratio to one of two sequence groups: Sequence 1, where participants received meal A in the first week and meal B in the second week; and sequence 2, where participants received meal B in the first week and meal A in the second week. Fifteen participants were allocated to each sequence group. 

The random number table was used to generate the allocation sequence. To ensure allocation concealment, an independent individual not affiliated with the study placed the random sequence into opaque, sealed envelopes. At baseline, each participant randomly selected one envelope, and the sequence inside determined the order in which they received the meals. Additionally, participants were blinded to the order of meal administration. Meals were prepared in advance to appear as identical as possible, with minimal distinguishing features, so that participants did not know which diet they were receiving at any stage. Standardization emphasized uniform meal presentation with minimal features to maintain participant blinding to the meal order. 

This study was a randomized controlled trial employing a crossover design and involving a total of 30 participants (15 women and 15 men). During the first phase, each participant received one of the two meal interventions: Either an HP-HF-LC meal or an HP-LF-HC meal. In the second phase, participants received the alternate meal. Due to the crossover design and the differences in food components between phases, blinding was feasible only for the sequence groups. 

### 4.2. Concealment Mechanism

Since participants consumed both intervention meals, the concealment applied only to the order in which the meals were administered.

### 4.3. Implementation

The registration of participants, generation of the allocation sequence, and assignment of the intervention type were managed by an individual who was independent of the research team. 

### 4.4. Data Collection and Management

#### 4.4.1. Blinding

Blinding is feasible only for the sequence groups.

#### 4.4.2. Procedure for Unblinding if Needed

Blinding is not applicable in this study.

### 4.5. Assessment and Outcomes

#### 4.5.1. Indirect Calorimetry Procedure

Participants began by resting in a supine position for 15 minutes to allow their heart rate to stabilize. After this period, a calorimetry mask was placed on their faces, and they breathed normally for 20 minutes. During this time, the indirect calorimetry device measured oxygen consumption and carbon dioxide production, enabling the calculation of resting energy expenditure, food oxidation rates, and respiratory rate. It was essential that participants remained awake, alert, and still throughout the calorimetry period. 

The indirect calorimetry measurements were conducted in five stages: Once before the intervention in a fasting state, and then at 1-, 2-, 3-, and 4-hours post-intervention. Due to the high sensitivity of indirect calorimetry, strict conditions were maintained to ensure maximum measurement accuracy. 

Calorimetry was performed in a separate, quiet room, isolated from other participants and distinct from the sampling area. Movements within the room were minimized, and the temperature was controlled between 22 and 24 degrees Celsius. Participants were required to fast for 10 - 12 hours before the start of the calorimetry measurement, with their evening meal being consistent with their usual diet. They were also advised to avoid strenuous physical activity on the day of the visit, arrive at the study center by vehicle, and refrain from smoking. 

#### 4.5.2. Appetite Evaluation

On the day of the study, participants completed a paper questionnaire at three key points: Before the intervention, one hour after the intervention, and at the conclusion of the intervention. This questionnaire assessed levels of satiety and hunger, with responses evaluated using a visual analog scale. To ensure consistency, participants’ attendance times were scheduled so that fasting did not exceed 12 hours. All measurements and evaluations were conducted between 7:00 am and 12:00 pm.

#### 4.5.3. Blood Sampling Protocol

Blood samples were collected in three phases: At baseline (fasting state), one hour after the intervention, and four hours after the intervention. The primary objective was to compare blood parameters at one hour and four hours post-intervention with those measured at the fasting baseline. The parameters to be analyzed included blood glucose, LDL, HDL, cholesterol, TG, TSH, T4, and epinephrine.

#### 4.5.4. Anthropometric Measurements

Anthropometric measurements were conducted at the beginning of each participant's visit. An experienced nutritionist at the research center was responsible for these evaluations to minimize measurement errors. To ensure accuracy, participants had to be confirmed as fasting and were instructed to avoid consuming liquids, including water, prior to the measurements. Whenever possible, participants were assessed with an empty bladder. Height was measured using a stadiometer with 1 mm accuracy, with participants standing barefoot. Weight was measured using a clinical scale with an accuracy of 100 g, with participants dressed in minimal clothing. Waist circumference was measured at the midpoint between the lower edge of the ribs and the top of the iliac crest, ensuring that the measuring tape was parallel to the ground. Abdominal circumference was measured horizontally at the level of the navel, and hip circumference was measured at the widest part of the buttocks. Body composition was analyzed using a bioelectrical impedance analyzer.

### 4.6. Monitoring and Adherence

To monitor and ensure adherence to the intervention protocol, five nutrition experts, well-versed in the study procedures, were involved at all stages. These experts oversaw anthropometric measurements, blood sampling, calorimetry, meal distribution, and participants’ resting periods. 

Participants were contacted three days prior to their scheduled visit to confirm the date and time. Additionally, they received a follow-up call the night before to review fasting requirements and attendance instructions. 

### 4.7. Data Management and Analysis

Data from anthropometric measurements, blood sampling, and calorimetry were recorded by the lead researchers using Microsoft Excel. Each participant's data was coded to ensure confidentiality, with only the principal researchers having access to identifying information. The coding system consisted of a three-digit number followed by letters A to E. The first digit (1 or 2) indicated the type of intervention, the next two digits denoted the participant number, and the letters represented the fasting stage and subsequent hours post-intervention. 

Data were reported as means ± standard deviations for continuous variables that followed a normal distribution, while non-normally distributed continuous variables were presented as medians along with interquartile ranges. Categorical variables were summarized using frequency distributions expressed as percentages. To compare conditions, independent two-sample *t*-tests were employed. Statistical analyses were conducted using generalized linear models (GLM) within SPSS version 22. The carryover effect was assessed, and if found significant, the influence of time and type of meal was analyzed. In the absence of a significant carryover effect, comparisons were made in parallel during the first period using covariance analysis. A two-factor repeated measures design was evaluated using generalized linear mixed models to examine the impact of time and type of meal across various phases of the study.

### 4.8. Collection, Evaluation and Storage of Blood Samples

A total of three blood samples were collected from each participant during the study. At each appointment, 5 cc of blood was drawn, with 2 cc designated for immediate evaluations. The remaining 3 cc was processed to separate the plasma, which was stored as a reserve for potential future analyses. 

To enhance the accuracy of laboratory measurements, the 2-cc blood samples from all participants were stored in a freezer at -22°C. After the final sample was collected from the last participant, all samples were analyzed together by a single operator using the same device and kit. At the end of each day, the 3-cc reserve samples were transferred directly to a -80°C freezer for long-term storage. 

### 4.9. Subgroup Analyses

Subgroup analyses were conducted to evaluate differences between male and female participants, as well as between two BMI categories (25 - 30 kg/m² and >30 kg/m²). Differences in BMI were adjusted for fat-free mass (FFM).

### 4.10. Non-adherence and Missing Data

Since this study was conducted during an acute phase without follow-up, missing data was not anticipated except in unforeseen circumstances. If a participant was unable to complete the study on the scheduled day, an attempt was made to reschedule their evaluation. If rescheduling was not possible, the participant was excluded from the study and replaced by another eligible individual.

### 4.11. Expected Outcomes

#### 4.11.1. Primary Outcomes

The primary expected outcomes included the results of five indirect calorimetry measurements for each participant at each stage, as well as assessments of satiety conducted before the intervention, one hour after, and four hours after the intervention.

#### 4.11.2. Secondary Outcomes

Secondary outcomes included measurements of blood glucose, insulin, LDL, HDL, cholesterol, TG, TSH, T4, and epinephrine levels in the fasting state, as well as one hour and four hours post-intervention. Additionally, blood pressure was recorded at both the beginning and the end of the intervention.

## 5. Discussion

Previous attempts to combat obesity have not yielded the expected results, contributing to the current epidemic, which has emerged as a global concern. The challenges associated with obesity are complex, extending beyond immediate health issues to impose a substantial burden on society and future generations (13). Generally, obesity and overweight conditions result from an imbalance between energy intake and expenditure. A common challenge in weight loss efforts is metabolic adaptation, a process in which the body’s basal metabolic rate decreases in response to reduced food intake and caloric consumption. Exploring strategies that reduce energy intake while preserving or enhancing energy expenditure may be critical for achieving effective weight reduction and ensuring long-term maintenance (6, 14). 

Research highlights the significant role of proteins in increasing energy expenditure and promoting satiety compared to carbohydrates and fats. It suggests that increasing the proportion of protein within total caloric intake can enhance feelings of fullness, even when meal sizes are reduced (15-17). However, there is ongoing debate about whether carbohydrates or fats have a greater effect on increasing metabolic rate, particularly RMR. 

The goal of this study is to identify a protein-rich dietary composition with varying fat and carbohydrate content that optimizes RMR while minimizing any negative impacts on basal metabolism. This approach could provide an effective strategy for managing overweight and obesity without adverse side effects.

Since this is a protocol study, the actual limitations will become more evident during the implementation phase. Nonetheless, some anticipated limitations include selection bias, as individuals volunteering for this study may differ in motivation or adherence compared to the general population. Reporting bias is another potential issue, as participants self-report their food intake, which may result in less accurate data. Additionally, generalization is limited to overweight and obese populations, as the study is specifically designed to address metabolic responses within these groups. Further studies will be needed to determine whether the outcomes are consistent in other populations. These challenges will be addressed following the study to ensure the results are presented with a robust perspective.

## Data Availability

Once the study is concluded and the results are published, access to the anonymized datasets will be granted upon reasonable request. However, approval to access the data will only be granted if all investigators consent to the request.

## Clinical Trial Registration Code:

Iranian Registry of Clinical Trials: IRCT20240106060633N1.
